# SNP-Associations and Phenotype Predictions from Hundreds of Microbial Genomes without Genome Alignments

**DOI:** 10.1371/journal.pone.0090490

**Published:** 2014-02-28

**Authors:** Barry G. Hall

**Affiliations:** Bellingham Research Institute, Bellingham, Washington, United States of America; Emory University School Of Medicine, United States of America

## Abstract

SNP-association studies are a starting point for identifying genes that may be responsible for specific phenotypes, such as disease traits. The vast bulk of tools for SNP-association studies are directed toward SNPs in the human genome, and I am unaware of any tools designed specifically for such studies in bacterial or viral genomes. The PPFS (Predict Phenotypes From SNPs) package described here is an add-on to ***kSNP***, a program that can identify SNPs in a data set of hundreds of microbial genomes. PPFS identifies those SNPs that are non-randomly associated with a phenotype based on the χ^2^ probability, then uses those diagnostic SNPs for two distinct, but related, purposes: (1) to predict the phenotypes of strains whose phenotypes are unknown, and (2) to identify those diagnostic SNPs that are most likely to be causally related to the phenotype. In the example illustrated here, from a set of 68 *E. coli* genomes, for 67 of which the pathogenicity phenotype was known, there were 418,500 SNPs. Using the phenotypes of 36 of those strains, PPFS identified 207 diagnostic SNPs. The diagnostic SNPs predicted the phenotypes of all of the genomes with 97% accuracy. It then identified 97 SNPs whose probability of being causally related to the pathogenic phenotype was >0.999. In a second example, from a set of 116 *E. coli* genome sequences, using the phenotypes of 65 strains PPFS identified 101 SNPs that predicted the source host (human or non-human) with 90% accuracy.

## Introduction

***kSNP*** [1], [2], is a program that identifies SNPs in bacterial and viral genomes (finished genomes, genome assemblies, or raw reads) without the necessity for genome alignment or reference genomes. *kSNP* v2 can analyze over 100 bacterial genomes in under 16 hours on a typical Linux cluster and in under 22 hours on a desktop Macintosh computer. *kSNP* has the further advantage of automatically generating Parsimony, Neighbor Joining, and Maximum Likelihood phylogenetic trees of the genomes. Several versions of the phylogenetic trees are provided, including those in which nodes are labeled with the number of SNPs that are found in all of the descendants of that node. kSNP automatically annotates the identified SNPs based upon the annotations in GenBank files. The number of SNPs that are identified can be enormous: over 100,000 SNPs from 288 genomes of the Rhabdoviridiae family, over 430,000 SNPS for 119 *Escherichia coli* genomes, over 1,388,000 SNPS in 207 Acinetobacter genomes [2].

SNP-association studies are the starting point for (1) identifying genes that may be responsible for specific phenotypes, such as disease traits, and (2) identifying potential gene-gene interactions. In bacteria and viruses they have the additional potential of identifying SNPs that might be incorporated into PCR probes for the purpose of quickly and inexpensively assessing phenotypes that are difficult, expensive or time consuming to determine by classical means, such as virulence or drug resistance in slow-growing organisms such as *Mycobacterium tuberculosis*.

Here I describe the PPFS package that identifies SNPs that are non-randomly associated with a phenotype. It is an “add on” to ***kSNP*** and has the following characteristics: (1) it runs seamlessly with ***kSNP***, (2) not all SNPs have to be present in all genomes, (3) not all genomes need to have a known phenotype, (4) it takes advantage of the fact that *kSNP* can provide annotations of most SNPS and incorporates those annotations into its analysis, (5) it is fast, requiring less than 10 minutes to analyze over 418,500 SNPs. The PPFS package of executables for Mac OS X and Linux operating systems, source code, and a detailed User Guide are freely available at https://sourceforge.net/projects/ppfs.

As a proof-of-principle I illustrate the uses of the PPFS package by identifying 207 SNPs associated with the phenotype “pathogenic” for a set of 68 *Escherichia coli* genomes. Pathogenic *E. coli* include EHEC (enterohemorrhagic), ETEC (enterotoxigenic) EPEC (enteropathogenic), ExPec (extraintestinal pathogenic), EAEC (entero-adherent), AIEC (adherent-invasive), UPEC (uropathogenic), and *Shigella*. Accession numbers, phenotypes and references for those phenotypes are included in Table S1A. I also show that when the phenotypes of only 36 of those genomes are known to the PPFS package, it predicts the phenotypes of the remaining strains with >94% accuracy. Finally, it identifies the 97 SNPs that are likely to be causally related to the pathogenic phenotype. In a second example, from a set of 116 *E. coli* genome sequences, using the phenotypes of 65 strains PPFS identified 101 SNPs that predicted the source host (human or non-human) with 90% accuracy. Accession numbers are included in Table S1B.

## Methods

The PPFS Package consists of five programs: ***PPFS, PickPhenotypeSubset, GetSNPprobs, DiagnosticSNPs*** and ***CausalSNPs***. It uses the output from *kSNP*, and must be run from with the *kSNP* output-files folder (directory). Using the full PPFS package requires that MEGA5 or MEGA 6 (available free at http://www.megasoftware.net/) be used to estimate ancestral states at internal nodes of an ML tree.

The user must provide a manually constructed file (.pheno file) in which the phenotype of each genome is listed either as positive (1), negative (0), or unknown (?).

***PPFS*** serves as a “front end” that takes the command line arguments, then runs ***PickPhenotypeSubset, GetSNPprobs,*** and ***DiagnosticSNPs*** without direct interaction with the user. After running those programs ***PPBS*** collects the output files of those programs into a folder (directory) named PPFS.

***PickPhenotypeSubset*** is used to select, from those genomes whose phenotypes are known, a random subset of genomes that will be used by *GetSNPprobs* to calculate, for each SNP, the probability that the alleles of that SNP are randomly associated with the phenotype. Through ***PPFS*** the user chooses the approximate fraction of strains to include in that subset. If the user sets the fraction to be included at 0.5, then for each strain whose phenotype is known in the.pheno file there is a 50% probability that its phenotype will be changed to unknown in the output subset.pheno file. That file thus provides a set of “unknown phenotype” genomes whose phenotype can be predicted by the *DiagnosticSNPs* program, allowing those predicted phenotype to be compared with the phenotypes in the.pheno file in order to assess the accuracy with which phenotypes are predicted.

***GetSNPprobs*** uses the output file from *PredictPhenotypeSubset* to calculate, for each SNP, the probability that the alleles of that SNP are randomly associated with the phenotype. Not all SNP sites are equally informative. For some sites the SNP alleles are distributed randomly with respect to the phenotype, with others the SNP alleles are biased. An extreme bias would be one in which all of the positive strains had an A and all of the negative strains had a G. A χ^2^ test is used to determine the probability that a SNP is distributed randomly with respect to phenotype.

Sites with low probabilities of the SNPs being randomly distributed with respect to phenotype are much more strongly correlated with the phenotype and have much more resolving power in predicting phenotypes than do sites with high probabilities. The χ^2^ probabilities of each SNP are written to an output file.

***GetSNPprobs*** writes two output files: (1) a.SNPs file that is the same as the input.fasta file except in a different format, and (2) a.ppcs (posterior probabilities chi-square) file. The.ppcs file lists, for each SNP, the probability that the alleles is distributed randomly with respect to the phenotype, and for each possible character state (ACGT-) the probability that a genome is positive and the probability that it is negative given that character state for that SNP.

***DiagnosticSNPs*** uses the original.pheno file, the subset.pheno file written by *PredictPhenotypeSubset*, and the output files written by *GetSNPprobs*, together with several files written by ***kSNP*** to identify the set of SNPs that most accurately predicts the phenotypes. Those SNPs are the “diagnostic SNPs”.

The SNPs are sorted according to the χ^2^ probability (*p*), then starting with the SNP with the lowest *p*, ***DiagnosticSNPs*** calculates the accuracy of the phenotype predictions. It then adds the next SNP in the sorted list and again calculates accuracy. A minimum of 50 SNPs are added, then SNP addition continues until one of several termination conditions is met: The accuracy with which positive strains are predicted (PPV) reaches a user-defined maximum (defaults to 0.98), or a user-defined number of SNPs is added (defaults to 1000), or the PPV (positive predictive value) declines below a user-defined fraction of the maximum PPV value so far (defaults to 0.97).

To understand the termination conditions it helps to understand the terms “accuracy”, “positive predictive value” (PPV) and “negative predictive value” (NPV). By comparing the known with predicted phenotypes the number of true positives (TP), True Negatives (TN), False Positive (FP) and False Negatives (FN) is determined. Accuracy is (TP+TN)/(TP+FP+TN+FN). Often we are actually more interested in knowing the probability that a strains that is predicted to be positive is actually positive. That probability is PPV, TP/(TP+FP). Similarly NPV, TN/(TN+FN), is the probability that a strain predicted to be negative is actually negative.

As SNPs with gradually increasing *p* values are added the PPV at first increases, but as *p* increases the quality of the SNPs for predictions declines, so that eventually a point is reached where addition of more lower-quality SNPs reduces the PPV. PPV does fluctuate somewhat, so addition is not terminated until PPV declines enough below its maximum value to be confident that the decline is real.

***DiagnosticSNPs*** writes several output files: (1) a DiagnosticSNPs.summary file (for an example see Table S2) that shows accuracy, PPV and NPV at each step as new SNPs are added; the final accuracy, PPV and NPV for those genomes whose phenotypes are known but were shown as unknown in the subset.pheno file; the predicted phenotype for each genome; and the accuracy, PPV and NPV averaged over all of the genomes. (2) a DiagnosticSNPs.call file (for an example see Table S3) that gives the predicted phenotype for each genome, but groups those predicted to be positive and those predicted to be negative. (3) a DiagnosticSNPs.list file that provides some annotation information for each diagnostic SNP (SNP ID, in a protein or not, synonymous or non-synonymous, protein accession number, and description of protein function). and (4) a DiagnosticSNPs.info file that provides the same information as the.list file but is restricted to non-synonymous substitution SNPs. (5 & 6) a SNPset.fasta and a SNPtranlations.txt file that are used by *CausalSNPs* and are discussed below.

***CausalSNPs*** identifies those diagnostic SNPs that are most likely to be causal with respect to the phenotype. Although predictive, the diagnostic SNPs may not be causally related to phenotype, and may only reflect evolutionary history. For instance, a SNP that arose along the same branch that a change in phenotype occurred may be perfectly associated with that phenotype but be completely unrelated to the reason that phenotype occurred. ***CausalSNPs*** is run from within the PPFS folder that was created by ***PPFS***.

It is often important to use SNPs to identify genes that are causally related to a phenotype. *CausalSNPs* is based on the assumption that diagnostic SNPs that are causally related to the phenotype are more likely than the average diagnostic SNP to change allele state along the same branches along which the phenotype changes. In order to determine the branches along which SNPs and the phenotype change it is necessary to infer the ancestral state of each SNP and of the phenotype at each internal node.

MEGA 5 [3] is used to estimate an ML tree from the SNP data, and then to calculate the ancestral state of each SNP at each internal node in order to identify the change in SNP allele across a branch from an ancestral to a descendant node. MEGA 5 cannot handle datasets with hundreds of genomes and hundreds of thousands of SNPs, so a reduced data set is written to the SNPset.fasta file by ***DiagnosticSNPs***. In that fasta file the sequence for each genome consists of the diagnostic SNPS, followed by a character that indicates the *predicted* phenotype of that genome, followed by a random sample of the SNPs. The number of random SNPs is 25 times the number of diagnostic SNPs, so if there are 100 diagnostic SNPs each sequence in the SNPset.fasta file will be 2601 characters, with the final 2500 having been randomly drawn from the original full set of SNPs. MEGA is run from within the PPFS folder that was created by PPFS.

In order to calculate ancestral states the ML tree estimated by MEGA must be rooted so that it has direction from ancestral to descendant nodes [4]. ML trees are unrooted, so by default MEGA roots the tree at its midpoint. Midpoint rooting assumes a molecular clock, an assumption that is often invalid [4]. It is far preferable to root the tree manually in MEGA using an outgroup genome. That outgroup may either be included in the kSNP analysis, or a separate KSNP analysis of the same data plus an outgroup can be used to produce an ML tree and that tree can be used to identify the branch along which the root should be manually placed in MEGA (virtual rooting). A brief protocol for estimating phylogenetic trees with MEGA 5 has recently been published [5], and an extensive and detailed description of its use is included in [4].

MEGA writes an output file of Most Probable Sequences that is used by *CausalSNPs*. See the PPFS User Guide for detailed instructions for using MEGA to estimate ancestral states from the SNPset.fasta file.

*CausalSNPs* parses the MEGA output file to identify the changes in the state of the allele of each diagnostic SNP across each branch, and to identify the change in phenotype across each branch. For each SNP *CausalSNPs* determines the χ^2^ probability *p* that the SNP changed randomly across the same branches across which the phenotype changed. It is necessary to consider the direction of the allele change in calculating those probabilities. For instance, if the phenotype changed from negative to positive across 10 branches, and the allele state of a particular diagnostic SNP changed along those same branches it could reasonably be inferred that the change in allele caused the change in phenotype. Howsoever, if five of those changes were from A to G, and the other 5 from G to A, then it would be unlikely that that SNP caused those changes. It is therefore necessary to classify each change as a “positive” or “negative” change. Changes from any base to the absence of the allele are considered negative, and base changes are considered positive if the value at the descendant node is greater than that at the ancestral node and negative if lower. The values of bases are A<C<G<T. The ordering of the values is arbitrary. Each SNP has only three possible states: one of two bases, or missing. For the purpose of calculating χ^2^ *p* values it is irrelevant whether a change from A to G is considered positive or negative, it only matters that the determination is consistent.

The diagnostic SNPs are ordered from lowest to highest *p*, with lower *p* values indicating a higher likelihood that the SNP is causal.

***CausalSNPs*** writes four output files that are collected into a folder (directory) named “CausalSNPs Output Files”:

1. CausalSNP.report (for an example see Table S4) gives the identities of the branches across which the phenotype changed and direction of those phenotype changes. It then gives, for each diagnostic SNP, the SNP number (the position of the SNP in the sequences in SNPset.fasta), the corresponding SNP ID in the kSNP files (the SNPtranslations.txt file written by *DiagnosticSNPs* keeps track of the SNP number and the corresponding SNP ID), the *p* that allele changes were random over the branches along which the phenotype changed, and whether the SNP is synonymous or non-synonymous, the protein accession number, and a brief description of the protein function.
2. A “ChiSquare_details.txt” file (for an example see Table S5) that gives for each SNP the observed and expected number of positive changes, negative changes, no changes, deletions, and additions of the SNP across the branches where the phenotype changed, the χ^2^ value, the degrees of freedom and *p*.
3. A “Changes.txt” file (for an example see Table S6) that give the change of each diagnostic SNP at each branch.
4. A “Branches.txt” file (for an example see Table S7) that gives the node number (as listed by MEGA) at each end of each branch.

### Genomes used in this Study

**Figure 1:**
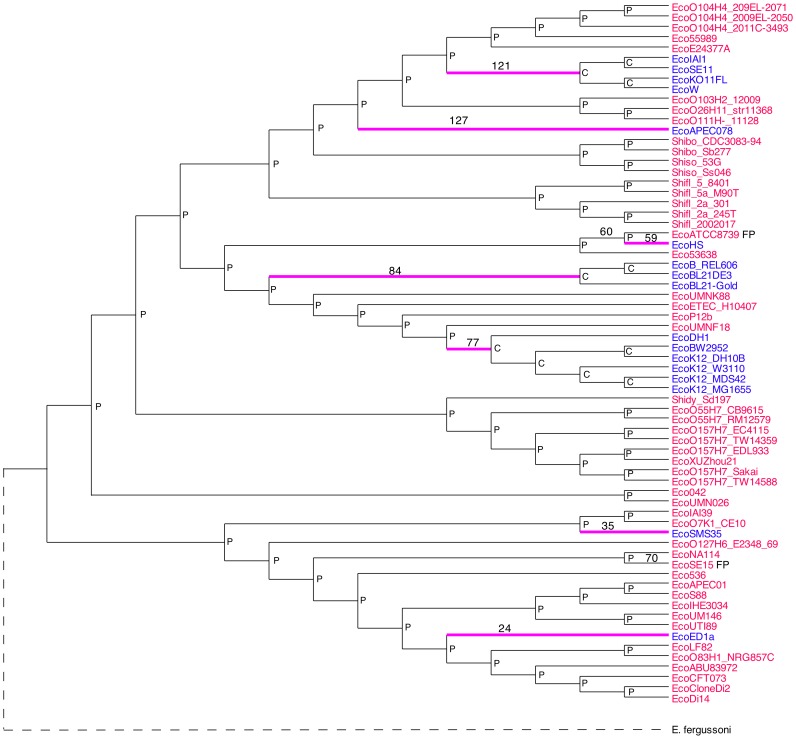
ML tree estimated by MEGA 5. The *predicted* phenotypes are indicated by the text color of the taxa: Red = pathogenic, Blue = commensal. The ancestral phenotypes are indicated by P (pathogenic) or C (commensal) at the internal nodes. Branches along which the phenotype changed are indicated in magenta. Numbers above some branches indicate the branch number given in Table S7. The dashed line indicates the virtual rooting with *Escherichia fergusonii*.

The Genbank accession numbers, corresponding ID on the tree in Figure 1, phenotype and reference for that phenotype are in Table S1.

## Results and Discussion

### SNP Analysis using kSNP

#### Data set 1

A set of 68 finished *E. coli* and *Shigella* genomes was analyzed on a Macintosh iMac computer with a 3.4 GHz Intel core i7 processor and with 16 GB of RAM as described in the User Guide to *kSNP*. All of those genomes were listed in a file of finished genome names, enabling *kSNP* to automatically retrieve and utilize the annotations for those genomes. The *kSNP* analysis required 10.3 hours, identified 418,500 SNPs, and is described in detail in [2].

In order to root the tree in MEGA *kSNP* was used to analyze the same 68 *E. coli* and *Shigella* genomes together with the finished genome of *Escherichia fergusonii* strain ATCC35469. Accession numbers, phenotypes and references for the genomes are in Table S1A of Supplementary Materials.

#### Data set 2

A set of 116 *Escherichia coli* genomes, including 8 finished genomes and 108 genome assemblies, was analyzed on the same Mac computer. The analysis required 15.6 hours and identified 470,806 SNPs. Accession numbers are in Table S1B.

### PPFS Analysis of Data Set 1 in which the Phenotypes are Pathogenic vs Commensal

In the file Eco68_patho.pheno human pathogenic strains were listed as the positive phenotype (1), others were listed as the negative phenotype (0), and one strain had an unknown phenotype. *PickPhenotypeSubsets* generated a subset of 36 strains whose phenotypes were given in the Eco68_patho_50%.pheno file (see Methods). That file also included 31 strains whose phenotype were changed to unknown in the Eco68_patho_50%.pheno file and that were used to calculate accuracies of phenotype predictions.

*GetSNPprobs* was used to calculate, for each of the 418,500 SNPS the χ^2^ probability that the alleles were distributed randomly with respect to phenotype.

*DiagnosticSNPs* identified 207 diagnostic SNPS that predicted the pathogenic phenotype for the 31 genomes whose phenotypes were unknown in the Eco68_patho_50%.pheno file. The accuracy of those predictions was 0.944, PPV was 0.931 and NPV was 1.0. Over all, of the 67 genomes whose phenotype is known, accuracy was 0.970, PPV was 0.96 and NPV was 1.0. Out of those 67 genomes there were two false positives (Table S3).

#### MEGA 5 analysis

An ML tree was estimated by MEGA 5 from the SNPset.fasta file of 5,384 SNPs per genome using the GTR+G model with 5 discrete Gamma categories. It was 94.5% topologically congruent with the ML tree estimated by *kSNP* from 418,500 SNPs as determined by the Compare2Trees web application (http://www.mas.ncl.ac.uk/~ntmwn/compare2trees/index.html) which is an updated version of the application reported in [6]. The tree was virtually rooted using *Escherichia fergusonii* strain ATCC35459, and ancestral states of the diagnostic SNPS and the phenotype were estimated (Fig. 1).

The analysis of ancestral phenotypes makes it clear that pathogenicity was the *E. coli* ancestral state. All seven changes were from pathogenic to commensal. Figure 1 suggests that pathogenicity is the ancestral state of *E. coli*, but that conclusion may be subject to sampling bias. Most of the sequenced *E. coli* strains were chosen specifically because they are pathogenic or because they are well known laboratory strains. Sequencing of a large number of commensals, including samples from healthy non-humans, plus environmental samples, would cast light on the concern with sampling bias.

*CausalSNPs* identified 97 SNPs whose probability of changing randomly across the branches was <0.001 (Table S4). That table lists the diagnostic SNPs in increasing order of *p*, the probability that the SNP allele changed randomly over the eight branches along which the pathogenicity phenotype changed. 1-*p* can be taken as the probability that the SNP is causally related to the pathogenicity phenotype.

The list of likely causal SNPs in Table S4 should not be treated as definitive, but should instead be read with some judgment. The first SNPs in that list, #81, is from a bacteriophage lambda tail assembly protein, for which no obvious role in pathogenicity leaps to mind. The next SNP, #8, is in a nitrogen assimilation protein, accession number CBG35053.1. Base changes at that SNP are synonymous, but examination of the χ^2^ details in Table S5 shows that on 6 of the 7 branches where phenotype changed the SNP change was deletion of that SNP. Furthermore, there were a total of nine diagnostic SNPs in that protein, all of which had *p* values <0.001. Similarly, the next SNP in the list, #26, is in a putative invasin, CBG35046.1. There are 10 diagnostic SNPs in that protein (Table S4), seven of which have *p* values <0.001. It appears quite reasonable to conclude that loss of invasin and the nitrogen assimilation protein are directly related to loss of pathogenicity.

#### Effects of false positives

Table S3 shows that there were two false positives, strains EcoATCC8739 and EcoSE15, indicated on Fig. 1 by appending FP to the genome ID. It is clear that the change from pathogenic to commensal phenotype occurred along branch 60, not branch 59; and that phenotype changed along branch 70 as well. Both false positives were strains that had been designated as unknown phenotype in the Eco68_patho_50%.pheno file. For such strains the accuracy was only 0.944. False positive and false negatives affect the correct assignment of the branches along which phenotypes change, and that in turn affects correct calculation of the χ^2^ probabilities. The reliability with which causal SNPs are identified is directly related to the accuracy with which “unknown” strains are called by the *DiagnosticSNPs* program. Undoubtedly, there will be many data sets for which the phenotypes of unknown strains cannot be called reliably. When the accuracy of those calls is <0.9 I do not suggest that identification of causal SNPs should be taken seriously.

The PPFS package helps identify SNPs that are associated with phenotypes, but does not attempt to detect interactions among those SNPs (epistasis). Multifactor Dimensionality Reduction (MDR) is a statistical approach to detecting such interactions [7] and is implemented by the free open-source MDR software package http://www.multifactordimensionalityreduction.org/. However “When you have more than 100 SNPs an exhaustive search may not be practical unless you are willing to wait days or even weeks for a run to finish. When the number of SNPs exceeds 10,000 an exhaustive search of all 3-way and higher combinations may be infeasible, even with a parallel computer.” [8] The PPFS package is a useful tool to reduce the number of SNPs to a level suitable for MDR analysis. MDR has a feature to downselect SNPs based on Chi-square probabilities, but it only considers core SNPs, those present in all strains, and only considers strains that have a phenotype, so does not make predictions about un-typed strains, limiting the SNPs and strains that can be included in an MDR analysis.

It is somewhat surprising that a phenotype as vague as “pathogenic” can be accurately predicted on the basis of a small set of diagnostic SNPs in a set of sequenced genomes. There may appear to be little point in predicting the phenotypes of completely sequenced genomes; after all, given the effort and expense of sequencing a microbial genome, the phenotype is likely to be known already. There are three reasons that phenotype prediction can be valuable today and is likely to become more valuable in the future. First, phenotypes are rarely reported in genome annotations and many genomes are submitted without reference to papers where those phenotypes might be available. The PPFS package permits prediction of the phenotypes of those genomes. Second, even when phenotypes are reported they are not necessarily the phenotype of interest to any particular investigator. Third, when phenotypes are difficult or time consuming to determine; e.g. for very slow growing organisms such as *M. tuberculosis* or for virulence phenotypes of viruses, PCR based assays can be designed to detect allele variants and thus to predict phenotypes. If a sufficient number of genome sequences are known to allow reliable genome predictions to be made, then those predictions can be applied to complete genomes, genome assemblies and raw-read genomes in the various databases. The cost of bacterial genome sequencing now approaches $100 US per genome and is likely to decrease significantly in the near future. It is quite feasible for a laboratory to characterize a phylogenetically diverse collection of a species with respect to a clinically important phenotype such as virulence, then to sequence 25–30 strains of each phenotype and to analyze those sequences with *kSNP* and the PPFS package. That analysis could then be used to predict the virulence of strains already in the database. If sequencing costs continue to decrease it may even become practical to routinely sequence genomes in epidemiological investigations. If the phenotype of interest is either expensive or time consuming to determine, the results of genome sequencing together with the information provided by a *kSNP*-PPFS analysis could provide a rapid assessment of the probability of exhibiting the clinically important phenotype.

### PPFS Analysis of Data Set 2 in which the Phenotypes are Human vs Non-human Host Source

*E. coli* is often used as a surrogate to indicate the presence of human pathogens in water resources [9]. When analyzing water resources it can be important to distinguish human (sewage, septic tank effluent, etc) source of contamination from non-human sources such as local fauna and agricultural resources (R. Norris and T. N. Fields, personal communication). Availability of DNA signatures for *E. coli* from human vs non-human sources would be valuable for distinguishing *E. coli* that indicate human waste contamination from those that are normally resident in soils near streams [10]. I have analyzed a set of 116 *E. coli* genomes in which the source host is specified in the annotations to determine whether there are SNPs that can predict human vs non-human origin.

In the Human.pheno file 61 strains were listed as the positive phenotype (1), i.e. coming from a human host, and 55 were listed as the negative (0) phenotype, i.e. coming from a non-human host. PickPhenotypeSubsets generated a subset of 65 strains whose phenotypes were given in the Human_50%.pheno file (see Methods). That file also included 51 strains whose phenotype were changed to unknown in theH_50%.pheno file and that were used to calculate accuracies of phenotype predictions. GetSNPprobs calculated, for each of the 470,806 SNPS the χ^2^ probability that the alleles were distributed randomly with respect to phenotype. DiagnosticSNPs identified 101 diagnostic SNPS that predicted the phenotype for the 51 genomes whose “source” phenotypes were unknown in the Human_50%.pheno file. The accuracy of those predictions was 0.845, PPV was 0.793 and NPV was 0.91. Over all, of the 116 genomes accuracy was 0.897, PPV was 0.877 and NPV was 0.923. Out of those 116 genomes there were seven false positives.

Although accuracy, PPV and NPV are not as high as in data set 1, for environmental sampling purposes where assessments would be based on determinations on many individual isolates, accuracies of about 90% are probably high enough to be useful.

## Conclusions

SNP association studies are not an end in themselves, but are a means to identify candidate genes for experimental studies related to the phenotype of interest. With the release of *kSNP* for SNP identification and the PPFS package for SNP association studies, the major barrier to SNP association studies in microorganisms is no longer the availability of appropriate tools; it is the culture of Genomic science itself. There are very few microorganisms for which both complete genome sequences and biological information are available in the databases. There are a plethora of databases that include or point to microbial genome sequences; e.g. NCBI's Genome database, SRA database, Bioprojects database, bacterial genome assembly database, but it is rare for any phenotypic information to be included in any of those files and most do not even refer to published papers that might shed light on phenotypes. The Broad Institute *E. coli* antibiotic resistance database http://www.broadinstitute.org/annotation/genome/escherichia_antibiotic_resistance/MultiHome.html provides detailed information on genotypes of hundreds of strains, but does not release the antibiotic resistance phenotypes. On the other side of the coin, the Network on Antimicrobial Resistance in *Staphylococcus aureus* (NARSA) http://www.narsa.net/control/member/allapprovedisolates provides antibiotic resistance phenotypes for hundreds of *S. aureus* strains, but genome sequences are available for only about a dozen of those strains. Genome sequencing has become so fast and so inexpensive that we seem to have lost track of the reasons for which those individual strains were sequenced. Obviously a complete description of the biological properties of each sequenced genome is impossible, but at the very least there should be a paragraph explaining why the particular strain was chosen to be sequenced. Was it from a sick or healthy individual, from an environmental sample (what environment), etc?

Ideally, an international repository would be created for maintenance of all strains (not strain information, but the organisms themselves) whose genomes are added to the genome sequence databases. Availability of the organisms would permit investigators to determine the phenotypes of interest of strains that have already been sequenced, and thus to conduct valuable SNP association studies. Failing that, the annotations of all genomes in the database should include contact information for an individual who is committed to providing a sample of the strain upon request.

## Supporting Information

Table S1 **GenBank accession numbers and literature citations for phenotypes of genomes in this study.**
(DOCX)

Table S2 **DiagnosticSNPs.summary file written by DiagnosticSNPs.**
(TXT)

Table S3 **DiagnosticSNPs.call file written by DiagnosticSNPs.**
(TXT)

Table S4 **CausalSNPs.report file written by CausalSNPs.**
(TXT)

Table S5 **Chi-square details.txt file written by CausalSNPs.**
(TXT)

Table S6 **Changes.txt file written by CausalSNPs.**
(TXT)

Table S7 **Branches.txt file written by CausalSNPs.**
(TXT)
