# Supplementary material for: SNP-Associations and Phenotype Predictions from Hundreds of Microbial Genomes without Genome Alignments
Source: PLoS One. 2014 Feb 28;9(2):e90490. doi: 10.1371/journal.pone.0090490 (PMC3938750; doi:10.1371/journal.pone.0090490)
Supplement: Table S1 — GenBank accession numbers and literature citations for phenotypes of genomes in this study. (DOCX) [file pone.0090490.s001.docx]

**Table S1 Strains, accession numbers and phenotypes**

**A. Pathogenic vs Commensal phenotypes**

| **Strain** | **ID on trees** | **Accession Number** | **Phenotype**  **and reference^a^** |
| --- | --- | --- | --- |
| **Finished Genomes** | | | |
| [Escherichia coli 042](http://www.ncbi.nlm.nih.gov/bioproject/40647) | Eco042 | [NC_017626.1](http://www.ncbi.nlm.nih.gov/nuccore/NC_017626.1) | EAEC [[1](#_ENREF_1)] |
| [Escherichia coli 536](http://www.ncbi.nlm.nih.gov/bioproject/16235) | Eco536 | NC_008253.1 | ExPec UTI [[2](#_ENREF_2)] |
| Escherichia coli 53638 | Eco53638 | AAKB00000000 | EIEC JCVI (http://gsc.jcvi.org/  projects/msc/e_coli_and_shigella  /escherichia_coli_53638  /index.shtml) |
| [Escherichia coli 55989](http://www.ncbi.nlm.nih.gov/bioproject/33413) | Eco55989 | [NC_011748.1](http://www.ncbi.nlm.nih.gov/nuccore/NC_011748.1) | EAEC [[3](#_ENREF_3)] |
| [Escherichia coli ABU 83972](http://www.ncbi.nlm.nih.gov/bioproject/38725) | EcoABU83972 | NC_017629.1 | ExPec UTI [[4](#_ENREF_4)] |
| [Escherichia coli APEC O1](http://www.ncbi.nlm.nih.gov/bioproject/16718) | EcoAPEC01 | [NC_008563.1](http://www.ncbi.nlm.nih.gov/nuccore/NC_008563.1) | ExPec From bird [[5](#_ENREF_5)] [[2](#_ENREF_2)] |
| Escherichia coli APEC 0[78](http://www.ncbi.nlm.nih.gov/bioproject/16718) | EcoAPEC078 | NC_020163.1 | APEC [[6](#_ENREF_6)] (pathogenic only to birds) |
| [Escherichia coli ATCC 8739](http://www.ncbi.nlm.nih.gov/bioproject/18083) | EcoATCC8739 | [NC_010468.1](http://www.ncbi.nlm.nih.gov/nuccore/NC_010468.1) | Commensal [[7](#_ENREF_7)] |
| [Escherichia coli B str. REL606](http://www.ncbi.nlm.nih.gov/bioproject/18281) | EcoB_REL606 | [NC_012967.1](http://www.ncbi.nlm.nih.gov/nuccore/NC_012967.1) | Commensal [[8](#_ENREF_8)] |
| [Escherichia coli 'BL21-Gold(DE3)pLysS AG'](http://www.ncbi.nlm.nih.gov/bioproject/30681) | EcoBL21-Gold | [NC_012947.1](http://www.ncbi.nlm.nih.gov/nuccore/NC_012947.1) | Commensal |
| [Escherichia coli BL21(DE3)](http://www.ncbi.nlm.nih.gov/bioproject/28965) | EcoBL21DE3 | [AM946981](http://www.ncbi.nlm.nih.gov/nuccore/AM946981) | Commensal [[8](#_ENREF_8)] |
| [Escherichia coli BW2952](http://www.ncbi.nlm.nih.gov/bioproject/33775) | EcoBW2952 | [NC_012759.1](http://www.ncbi.nlm.nih.gov/nuccore/NC_012759.1) | Commensal [[9](#_ENREF_9)] |
| Escherichia coli CFT073 | EcoCFT073 | [NC_004431.1](http://www.ncbi.nlm.nih.gov/nuccore/NC_004431.1) | ExPec [[2](#_ENREF_2)] |
| Escherichia coli str. 'clone D i2' | EcoCloneDi2 | CP002212 | ExPec UPEC [[10](#_ENREF_10)] |
| Escherichia coli str. 'clone D i14' | EcoCloneDi14 | CP002212 | ExPec UPEC [[10](#_ENREF_10)] |
| Escherichia coli DH1 | EcoDH1 | [CP001637](http://www.ncbi.nlm.nih.gov/nuccore/CP001637) | Commensal [[11](#_ENREF_11)] |
| Escherichia coli E24377A | EcoE24377A | [NC_009801.1](http://www.ncbi.nlm.nih.gov/nuccore/NC_009801.1) | ETEC |
| Escherichia coli ED1a | EcoED1a | NC_011745.1 | Commensal [[2](#_ENREF_2)] |
| Escherichia coli ETEC H10407 | EcoETEC_H10407 | FN649414 | ETEC [[2](#_ENREF_2)] |
| Escherichia coli HS | EcoHS | NC_009800.1 | Commensal [[2](#_ENREF_2)] |
| Escherichia coli IAI1 | EcoIAI1 | NC_011741.1 | Commensal [[2](#_ENREF_2)] |
| Escherichia coli IAI39 | EcoIAI39 | NC_011750.1 | ExPec UPEC [[2](#_ENREF_2), [12](#_ENREF_12)] |
| Escherichia coli IHE3034 | EcoIHE3034 | CP001969 | ExPec Neonatal meningitis [[13](#_ENREF_13)] |
| Escherichia coli str. K-12 substr. DH10B | EcoK12_DH10B | NC_010473.1 | Commensal [[2](#_ENREF_2)] |
| Escherichia coli str. K-12 substr. MDS42 | EcoK12_MDS42 | NC_020518.1 | Commensal |
| Escherichia coli str. K-12 substr. MG1655 | EcoK12_MG1655 | NC_000913.2 | Commensal [[3](#_ENREF_3)] |
| Escherichia coli str. K-12 substr. W3110 | EcoK12_W3110 | AP009048 | Commensal [[8](#_ENREF_8)] |
| Escherichia coli KO11FL | EcoKO11FL | CP002516 | Commensal [[14](#_ENREF_14)] |
| Escherichia coli LF82 | EcoLF82 | CU651637 | AIEC [[15](#_ENREF_15)] |
| Escherichia coli NA114 | EcoNA114 | CP002797 | ExPec UPEC [[16](#_ENREF_16)] |
| Escherichia coli O103:H2 str. 12009 | EcoO103H2_12009 | NC_013353.1 | EHEC [[8](#_ENREF_8)] |
| Escherichia coli O104:H4 2009EL-2050 | EcoO104H4_2009EL-2050 | NC_018650 | EAEC/STEC [[17](#_ENREF_17)] |
| Escherichia coli O104:H4 2009EL-2071 | EcoO104H4_2009EL-2071 | NC_018661 | EAEC/STEC [[17](#_ENREF_17)] |
| Escherichia coliO104:H4 2011C-3493 | EcoO104H4_2011C-3493 | NC_018658 | EAEC/STEC [[17](#_ENREF_17)] |
| Escherichia coli O111:H- str. 11128 | EcoO111H-_11128 | NC_013364.1 | EHEC [[8](#_ENREF_8)] |
| Escherichia coli O127:H6 str. E2348/69 | EcoO127H6_E2348_ | NC_011601.1 | EPEC [[2](#_ENREF_2)] |
| Escherichia coli O157:H7 str. EC4115 | EcoO157H7_EC4115 | NC_011353.1 | EHEC [[8](#_ENREF_8)] |
| Escherichia coli O157:H7 str. EDL933 | EcoO157H7_EDL933 | NC_002655.2 | EHEC [[8](#_ENREF_8)] |
| Escherichia coli O157:H7 str. Sakai | EcoO157H7_Sakai | NC_002695.1 | EHEC [[8](#_ENREF_8)] |
| Escherichia coli O157:H7 str. TW14359 | EcoO157H7_TW14359 | NC_013008.1 | EHEC [[8](#_ENREF_8)] |
| Escherichia coli O157H7 str. TW14588 | EcoO157H7_TW14588 | [CM000662.1](http://www.ncbi.nlm.nih.gov/nuccore/CM000662.1) | EHEC [[18](#_ENREF_18)] |
| Escherichia coli O26:H11 str. 11368 | EcoO26H11_str11368 | NC_013361.1 | EHEC [[8](#_ENREF_8)] |
| Escherichia coli O55:H7 str. CB9615 | EcoO55H7_CB9615 | NC_013941.1 | EPEC [[2](#_ENREF_2)] |
| Escherichia coli O55:H7 str. RM12579 | EcoO55H7_RM12579 | NC_017653 | EPEC [[19](#_ENREF_19)] |
| Escherichia coli O7:K1 str. CE10 | EcoO7K1_CE10 | NC_017646 | ExPec Neonatal meningitis Lu et al 2011 |
| Escherichia coli O83:H1 str. NRG 857C | EcoO83H1_NRG857C | CP001855 | AIEC [[20](#_ENREF_20)] |
| [Escherichia coli P12b](http://www.ncbi.nlm.nih.gov/genome/167?project_id=162061) | EcoP21B | [CP002291.1](http://www.ncbi.nlm.nih.gov/nuccore/CP002291.1) | ????? [[21](#_ENREF_21)] |
| Escherichia coli S88 | EcoS88 | NC_011742.1 | ExPec Neonatal Meningitis [[12](#_ENREF_12)] [[2](#_ENREF_2)] |
| Escherichia coli SE11 | EcoSE11 | NC_011415.1 | Commensal [[2](#_ENREF_2)] |
| Escherichia coli SE15 | EcoSE15 | AP009378 | Commensal [[22](#_ENREF_22)] |
| Escherichia coli SMS-3-5 | EcoSMS35 | NC_010498.1 | Commensal [[2](#_ENREF_2)] |
| Escherichia coli UM146 | EcoUM146 | CP002167 | AIEC, Ileal Chron's Disease biopsy tissue [[23](#_ENREF_23)] |
| Escherichia coli UMN026 | EcoUMN026 | NC_011751.1 | ExPec UPEC [[12](#_ENREF_12)] [[2](#_ENREF_2)] |
| Escherichia coli UMNF18 | EcoUMNF18 | NZ_AGTD01000001.1 | ETEC ([[24](#_ENREF_24)]) |
| Escherichia coli UMNK88 | EcoUMNK88 | NC_017639 | ETEC [[24](#_ENREF_24), [25](#_ENREF_25)] |
| Escherichia coli UTI89 | EcoUTI89 | NC_007946.1 | ExPec UPEC [[2](#_ENREF_2)] |
| Escherichia coli W | EcoW | CP002185 | Commensal |
| Escherichia coli Xuzhou21 | EcoXUZhou21 | \|  \| CP001925.1 \| \| --- \| --- \| | EHEC [[26](#_ENREF_26)] |
| Shigella boydii CDC 3083-94 | Shibo_CDC3083-94 | NC_010658.1 | [[27](#_ENREF_27)] |
| Shigella boydii Sb227 | Shibo_Sb277 | NC_007613.1 | [[28](#_ENREF_28)] |
| Shigella dysenteriae Sd197 | Shidy_Sd197 | NC_007606.1 | [[28](#_ENREF_28)] |
| Shigella flexneri 2002017 | Shifl_2002017 | NC_004741.1 | [[29](#_ENREF_29)] |
| Shigella flexneri 2a str. 2457T | Shifl_2a_245T | NC_004741.1 | [[30](#_ENREF_30)] |
| Shigella flexneri 2a str. 301 | Shifl_2a_301 | NC_004337.2 | [[31](#_ENREF_31)] |
| Shigella flexneri 5 str. 8401 | Shifl_5_8401 | NC_008258.1 | [[32](#_ENREF_32)] |
| Shigella flexneri 5a str. M90T | Shifi_M90T | CM001474.1 | [[33](#_ENREF_33)] |
| Shigella sonnei Ss046 | Shiso_Ss046 | NC_008258.1 | [[28](#_ENREF_28)] |
| Shigella sonnei 53G | Shiso_53G | HE616528.1 |  |
| Escherichia fergusonii ATCC35469 | E. fergusonii | NC_011740.1 |  |

^a^If no reference is given the genome is a direct submission and the phenotype is taken from the GenBank file annotation

**B. Human vs non-human source**

| **Strain** | **Accession number** | **Source (host)^a^** |
| --- | --- | --- |
| Eco_9.0111 | NZ_AEZZ00000000 | Homo sapiens |
| Eco_93.0624 | NZ_AEZT00000000 | Homo sapiens |
| Eco_95.0941 | NZ_AEZN00000000 | Homo sapiens |
| Eco_96.0497 | NZ_AEZQ00000000 | Homo sapiens |
| Eco_96.154 | NZ_AEZW00000000 | Homo sapiens |
| Eco_DEC10A | NZ_AIGP00000000 | Homo sapiens |
| Eco_DEC10B | NZ_AIGQ00000000 | Homo sapiens |
| Eco_DEC10C | NZ_AIGR00000000 | Homo sapiens |
| Eco_DEC10D | NZ_AIGS00000000 | Homo sapiens |
| Eco_DEC11A | NZ_AIGV00000000 | Homo sapiens |
| Eco_DEC11B | NZ_AIGW00000000 | Homo sapiens |
| Eco_DEC11C | NZ_AIGX00000000 | Homo sapiens |
| Eco_DEC11D | NZ_AIGY00000000 | Homo sapiens |
| Eco_DEC11E | NZ_AIGZ00000000 | Homo sapiens |
| Eco_DEC12A | NZ_AIHA00000000 | Homo sapiens |
| Eco_DEC12B | NZ_AIHB00000000 | Homo sapiens |
| Eco_DEC12C | NZ_AIHC00000000 | Homo sapiens |
| Eco_DEC12D | NZ_AIHD00000000 | Homo sapiens |
| Eco_DEC12E | NZ_AIHE00000000 | Homo sapiens |
| Eco_DEC13A | NZ_AIHF00000000 | Homo sapiens |
| Eco_DEC13B | NZ_AIHG00000000 | Homo sapiens |
| Eco_DEC13C | NZ_AIHH00000000 | Homo sapiens |
| Eco_DEC13D | NZ_AIHI00000000 | Homo sapiens |
| Eco_DEC13E | NZ_AIHJ00000000 | Homo sapiens |
| Eco_DEC14A | NZ_AIHK00000000 | Homo sapiens |
| Eco_DEC14B | NZ_AIHL00000000 | Homo sapiens |
| Eco_DEC14C | NZ_AIHM00000000 | Homo sapiens |
| Eco_DEC14D | NZ_AIHN00000000 | Homo sapiens |
| Eco_DEC15A | NZ_AIHO00000000 | Homo sapiens |
| Eco_DEC15B | NZ_AIHP00000000 | Homo sapiens |
| Eco_DEC15C | NZ_AIHQ00000000 | Homo sapiens |
| Eco_DEC15D | NZ_AIHR00000000 | Homo sapiens |
| Eco_DEC15E | NZ_AIHS00000000 | Homo sapiens |
| Eco_DEC1A | NZ_AIEV00000000 | Homo sapiens |
| Eco_DEC1B | NZ_AIEW00000000 | Homo sapiens |
| Eco_DEC1C | NZ_AIEX00000000 | Homo sapiens |
| Eco_DEC1D | NZ_AIEY00000000 | Homo sapiens |
| Eco_DEC1E | NZ_AIEZ00000000 | Homo sapiens |
| Eco_DEC2A | NZ_AIFA00000000 | Homo sapiens |
| Eco_DEC2B | NZ_AFJB00000000 | Homo sapiens |
| Eco_DEC2C | NZ_AIFB00000000 | Homo sapiens |
| Eco_DEC2D | NZ_AIFC00000000 | Homo sapiens |
| Eco_DEC2E | NZ_AIFD00000000 | Homo sapiens |
| Eco_DEC3A | NZ_AIFE00000000 | Homo sapiens |
| Eco_DEC3B | NZ_AIFF00000000 | Homo sapiens |
| Eco_DEC3C | NZ_AIFG00000000 | Homo sapiens |
| Eco_DEC3D | NZ_AIFH00000000 | Homo sapiens |
| Eco_DEC3E | NZ_AIFI00000000 | Homo sapiens |
| Eco_DEC3F | NZ_AIFJ00000000 | Homo sapiens |
| Eco_DEC4B | NZ_AIFL00000000 | Homo sapiens |
| Eco_DEC4E | NZ_AIFO00000000 | Homo sapiens |
| Eco_DEC5A | NZ_AIFQ00000000 | Homo sapiens |
| Eco_DEC5B | NZ_AIFR00000000 | Homo sapiens |
| Eco_DEC5C | NZ_AIFS00000000 | Homo sapiens |
| Escherichia_coli_0.1288_425420599 | NZ_AMVJ00000000 | Cow |
| Escherichia_coli_08BKT055439_545140219 | NZ_AVRI00000000 | Cow |
| Escherichia_coli_08BKT77219_545140599 | NZ_AVRJ00000000 | Cow |
| Escherichia_coli_09BKT024447_545141062 | NZ_AVRK00000000 | Cow |
| Escherichia_coli_09BKT076207_545136309 | NZ_AVQR00000000 | Cow |
| Escherichia_coli_1.2264_417152015 | NZ_AEZO00000000 | goat |
| Escherichia_coli_1.2741_417119824 | NZ_AEZI00000000 | Cow |
| Escherichia_coli_3.2608_417178633 | NZ_AEZS00000000 | Horse |
| Escherichia_coli_3.3884_417270398 | NZ_AFAC00000000 | Cow |
| Escherichia_coli_3.4880_445048619 | NZ_AOET00000000 | Cow |
| Escherichia_coli_4.0522_417203081 | NZ_AEZU00000000 | Cow |
| Escherichia_coli_4.0967_417254869 | NZ_AFAA00000000 | Rabbit |
| Escherichia_coli_5.0588_417136359 | NZ_AEZK00000000 | Cow |
| Escherichia_coli_7.1982_445026466 | NZ_AOEP00000000 | Cow |
| Escherichia_coli_9.1649_452339940 | NZ_AEZY00000000 | Pig |
| Escherichia_coli_900105_417300141 | NZ_AFAI00000000 | Cow |
| Escherichia_coli_97.0246_417129236 | NZ_AEZJ00000000 | Cow |
| Escherichia_coli_97.0264_452335920 | NZ_AEZP00000000 | Cow |
| Escherichia_coli_99.0670_445061729 | NZ_AOEV00000000 | Cow |
| Escherichia_coli_99.0678_429076139 | NZ_ANME00000000 | Cow |
| Escherichia_coli_99.0713_429081522 | NZ_ANMF00000000 | Cow |
| Escherichia_coli_99.0741_417170723 | NZ_AEZR00000000 | Cow |
| Escherichia_coli_AA86_417665128 | NZ_AFET00000000 | Cow |
| Escherichia_coli_AD30_421779006 | NZ_AMSK00000000 | Chicken |
| Escherichia_coli_B41_417294281 | NZ_AFAH00000000 | Pig |
| Escherichia_coli_Bd5610_99_545141564 | NZ_AVRL00000000 | Cow |
| Escherichia_coli_CUMT8_419952369 | NZ_AJWV00000000 | Mouse |
| Escherichia_coli_DEC10E_419281345 | NZ_AIGT00000000 | Cow |
| Escherichia_coli_DEC10F_419287513 | NZ_AIGU00000000 | Rabbit |
| Escherichia_coli_DEC4A_419084069 | NZ_AIFK00000000 | Cow |
| Escherichia_coli_DEC4C_419095954 | NZ_AIFM00000000 | Bison |
| Escherichia_coli_DEC4D_419101574 | NZ_AIFN00000000 | Cow |
| Escherichia_coli_DEC7A_419173376 | NZ_AIGA00000000 | Pig |
| Escherichia_coli_DEC7B_419178898 | NZ_AIGB00000000 | Cow |
| Escherichia_coli_DEC7C_419184295 | NZ_AIGC00000000 | Pig |
| Escherichia_coli_DEC7D_419189743 | NZ_AIGD00000000 | Pig |
| Escherichia_coli_DEC7E_419194916 | NZ_AIGE00000000 | Pig |
| Escherichia_coli_DEC8C_419213257 | NZ_AIGH00000000 | Cow |
| Escherichia_coli_FRIK920_421827501 | NZ_AMTP00000000 | Cow |
| Escherichia_coli_KD1_419916220 | NZ_AJWO00000000 | Dog |
| Escherichia_coli_O08_450237527 | NZ_AOGM00000000 | Chicken |
| Escherichia_coli_O157_478764724 | NZ_AHZD00000000 | Cow |
| Escherichia_coli_O32_419813582 | NZ_AJQW00000000 | Cow |
| Escherichia_coli_S17_450262410 | NZ_AOGN00000000 | Chicken |
| Escherichia_coli_SEPT362_450208582 | NZ_AOGL00000000 | Chicken |
| Escherichia_coli_SWW33_487679359 | NZ_AQFX00000000 | Mouse |
| Escherichia_coli_T1282_01_545141930 | NZ_AVRM00000000 | Cow |
| Escherichia_coli_T1840_97_545142167 | NZ_AVRN00000000 | Cow |
| Escherichia_coli_T234_00_545142500 | NZ_AVRO00000000 | Cow |
| Escherichia_coli_T924_01_545142806 | NZ_AVRP00000000 | Cow |
| Escherichia_coli_Tx1686_545148044 | NZ_AVSN00000000 | Cow |
| Escherichia_coli_Tx3800_545148289 | NZ_AVSO00000000 | Cow |
| Escherichia_coli_UMNF18_418306523 | NZ_AGTD00000000 | Pig |
| Escherichia_coli_W26_418044527 | NZ_AGIA00000000 | Cow |
| Escherichia_coli_O55:H7_str_CB9615 | NC_013941.1 | Homo sapiens |
| Escherichia_coli_ABU 83972 | NC_017629 | Homo sapiens |
| Escherichia_coli_UMNK88 | NC_017639 | Pig |
| Escherichia_coli_O7:K1_CE10 | NC_017646 | Homo sapiens |
| Escherichia_coli_O55:H7_str_RM12579 | NC_017653 | Homo sapiens |
| Escherichia_coli_O104:H4_str_2009EL-2071 | NC_0186614 | Homo sapiens |
| Escherichia_coli_O104:H4_str_2009EL-2050 | NC_018650 | Homo sapiens |
| Escherichia_coli_O104:H4_str_2011C-3493 | NC_018658 | Homo sapiens |

^a^The source (host) of each strain is taken from the GenBank annotations.

**References**

1. Crossman LC, Chaudhuri RR, Beatson SA, Wells TJ, Desvaux M, Cunningham AF, Petty NK, Mahon V, Brinkley C, Hobman JL *et al*: **A commensal gone bad: complete genome sequence of the prototypical enterotoxigenic Escherichia coli strain H10407**. *Journal of bacteriology* 2010, **192**(21):5822-5831.

2. Zhou Z, Li X, Liu B, Beutin L, Xu J, Ren Y, Feng L, Lan R, Reeves PR, Wang L: **Derivation of Escherichia coli O157:H7 from its O55:H7 precursor**. *PloS one* 2010, **5**(1):e8700.

3. Sims GE, Kim SH: **Whole-genome phylogeny of Escherichia coli/Shigella group by feature frequency profiles (FFPs)**. *Proc Natl Acad Sci U S A* 2011, **108**(20):8329-8334.

4. Zdziarski J, Brzuszkiewicz E, Wullt B, Liesegang H, Biran D, Voigt B, Gronberg-Hernandez J, Ragnarsdottir B, Hecker M, Ron EZ *et al*: **Host imprints on bacterial genomes--rapid, divergent evolution in individual patients**. *PLoS pathogens* 2010, **6**(8):e1001078.

5. Johnson TJ, Kariyawasam S, Wannemuehler Y, Mangiamele P, Johnson SJ, Doetkott C, Skyberg JA, Lynne AM, Johnson JR, Nolan LK: **The genome sequence of avian pathogenic Escherichia coli strain O1:K1:H7 shares strong similarities with human extraintestinal pathogenic E. coli genomes**. *Journal of bacteriology* 2007, **189**(8):3228-3236.

6. Mangiamele P, Nicholson B, Wannemuehler Y, Seemann T, Logue CM, Li G, Tivendale KA, Nolan LK: **Complete genome sequence of the avian pathogenic Escherichia coli strain APEC O78**. *Genome announcements* 2013, **1**(2):e0002613.

7. Archer CT, Kim JF, Jeong H, Park JH, Vickers CE, Lee SY, Nielsen LK: **The genome sequence of E. coli W (ATCC 9637): comparative genome analysis and an improved genome-scale reconstruction of E. coli**. *BMC Genomics* 2011, **12**:9.

8. Sims GE, Kim SH: **Whole-genome phylogeny of Escherichia coli/Shigella group by feature frequency profiles (FFPs)**. *Proceedings of the National Academy of Sciences of the United States of America* 2011, **108**(20):8329-8334.

9. Ferenci T, Zhou Z, Betteridge T, Ren Y, Liu Y, Feng L, Reeves PR, Wang L: **Genomic sequencing reveals regulatory mutations and recombinational events in the widely used MC4100 lineage of Escherichia coli K-12**. *Journal of bacteriology* 2009, **191**(12):4025-4029.

10. Reeves PR, Liu B, Zhou Z, Li D, Guo D, Ren Y, Clabots C, Lan R, Johnson JR, Wang L: **Rates of mutation and host transmission for an Escherichia coli clone over 3 years**. *PloS one* 2011, **6**(10):e26907.

11. Suzuki S, Ono N, Furusawa C, Ying BW, Yomo T: **Comparison of sequence reads obtained from three next-generation sequencing platforms**. *PloS one* 2011, **6**(5):e19534.

12. Touchon M, Hoede C, Tenaillon O, Barbe V, Baeriswyl S, Bidet P, Bingen E, Bonacorsi S, Bouchier C, Bouvet O *et al*: **Organised genome dynamics in the Escherichia coli species results in highly diverse adaptive paths**. *PLoS genetics* 2009, **5**(1):e1000344.

13. Moriel DG, Bertoldi I, Spagnuolo A, Marchi S, Rosini R, Nesta B, Pastorello I, Corea VA, Torricelli G, Cartocci E *et al*: **Identification of protective and broadly conserved vaccine antigens from the genome of extraintestinal pathogenic Escherichia coli**. *Proc Natl Acad Sci U S A* 2010, **107**(20):9072-9077.

14. Turner PC, Yomano LP, Jarboe LR, York SW, Baggett CL, Moritz BE, Zentz EB, Shanmugam KT, Ingram LO: **Optical mapping and sequencing of the Escherichia coli KO11 genome reveal extensive chromosomal rearrangements, and multiple tandem copies of the Zymomonas mobilis pdc and adhB genes**. *Journal of industrial microbiology & biotechnology* 2012, **39**(4):629-639.

15. Wine E, Ossa JC, Gray-Owen SD, Sherman PM: **Adherent-invasive Escherichia coli, strain LF82 disrupts apical junctional complexes in polarized epithelia**. *BMC microbiology* 2009, **9**:180.

16. Avasthi TS, Kumar N, Baddam R, Hussain A, Nandanwar N, Jadhav S, Ahmed N: **Genome of multidrug-resistant uropathogenic Escherichia coli strain NA114 from India**. *Journal of bacteriology* 2011, **193**(16):4272-4273.

17. Ahmed SA, Awosika J, Baldwin C, Bishop-Lilly KA, Biswas B, Broomall S, Chain PS, Chertkov O, Chokoshvili O, Coyne S *et al*: **Genomic comparison of Escherichia coli O104:H4 isolates from 2009 and 2011 reveals plasmid, and prophage heterogeneity, including shiga toxin encoding phage stx2**. *PloS one* 2012, **7**(11):e48228.

18. Kulasekara BR, Jacobs M, Zhou Y, Wu Z, Sims E, Saenphimmachak C, Rohmer L, Ritchie JM, Radey M, McKevitt M *et al*: **Analysis of the genome of the Escherichia coli O157:H7 2006 spinach-associated outbreak isolate indicates candidate genes that may enhance virulence**. *Infection and immunity* 2009, **77**(9):3713-3721.

19. Kyle JL, Cummings CA, Parker CT, Quinones B, Vatta P, Newton E, Huynh S, Swimley M, Degoricija L, Barker M *et al*: **Escherichia coli serotype O55:H7 diversity supports parallel acquisition of bacteriophage at Shiga toxin phage insertion sites during evolution of the O157:H7 lineage**. *Journal of bacteriology* 2012, **194**(8):1885-1896.

20. Allen CA, Niesel DW, Torres AG: **The effects of low-shear stress on Adherent-invasive Escherichia coli**. *Environmental microbiology* 2008, **10**(6):1512-1525.

21. Liu B, Hu B, Zhou Z, Guo D, Guo X, Ding P, Feng L, Wang L: **A novel non-homologous recombination-mediated mechanism for Escherichia coli unilateral flagellar phase variation**. *Nucleic Acids Res* 2012, **40**(10):4530-4538.

22. Toh H, Oshima K, Toyoda A, Ogura Y, Ooka T, Sasamoto H, Park SH, Iyoda S, Kurokawa K, Morita H *et al*: **Complete genome sequence of the wild-type commensal Escherichia coli strain SE15, belonging to phylogenetic group B2**. *Journal of bacteriology* 2010, **192**(4):1165-1166.

23. Krause DO, Little AC, Dowd SE, Bernstein CN: **Complete genome sequence of adherent invasive Escherichia coli UM146 isolated from Ileal Crohn's disease biopsy tissue**. *Journal of bacteriology* 2011, **193**(2):583.

24. Shepard SM, Danzeisen JL, Isaacson RE, Seemann T, Achtman M, Johnson TJ: **Genome sequences and phylogenetic analysis of K88- and F18-positive porcine enterotoxigenic Escherichia coli**. *Journal of bacteriology* 2012, **194**(2):395-405.

25. Fernandez-Alarcon C, Singer RS, Johnson TJ: **Comparative genomics of multidrug resistance-encoding IncA/C plasmids from commensal and pathogenic Escherichia coli from multiple animal sources**. *PloS one* 2011, **6**(8):e23415.

26. Xiong Y, Wang P, Lan R, Ye C, Wang H, Ren J, Jing H, Wang Y, Zhou Z, Bai X *et al*: **A novel Escherichia coli O157:H7 clone causing a major hemolytic uremic syndrome outbreak in China**. *PloS one* 2012, **7**(4):e36144.

27. Pupo GM, Lan R, Reeves PR: **Multiple independent origins of Shigella clones of Escherichia coli and convergent evolution of many of their characteristics**. *Proc Natl Acad Sci U S A* 2000, **97**(19):10567-10572.

28. Yang F, Yang J, Zhang X, Chen L, Jiang Y, Yan Y, Tang X, Wang J, Xiong Z, Dong J *et al*: **Genome dynamics and diversity of Shigella species, the etiologic agents of bacillary dysentery**. *Nucleic Acids Res* 2005, **33**(19):6445-6458.

29. Ye C, Lan R, Xia S, Zhang J, Sun Q, Zhang S, Jing H, Wang L, Li Z, Zhou Z *et al*: **Emergence of a new multidrug-resistant serotype X variant in an epidemic clone of Shigella flexneri**. *J Clin Microbiol* 2010, **48**(2):419-426.

30. Wei J, Goldberg MB, Burland V, Venkatesan MM, Deng W, Fournier G, Mayhew GF, Plunkett G, 3rd, Rose DJ, Darling A *et al*: **Complete genome sequence and comparative genomics of Shigella flexneri serotype 2a strain 2457T**. *Infection and immunity* 2003, **71**(5):2775-2786.

31. Jin Q, Yuan Z, Xu J, Wang Y, Shen Y, Lu W, Wang J, Liu H, Yang J, Yang F *et al*: **Genome sequence of Shigella flexneri 2a: insights into pathogenicity through comparison with genomes of Escherichia coli K12 and O157**. *Nucleic Acids Res* 2002, **30**(20):4432-4441.

32. Nie H, Yang F, Zhang X, Yang J, Chen L, Wang J, Xiong Z, Peng J, Sun L, Dong J *et al*: **Complete genome sequence of Shigella flexneri 5b and comparison with Shigella flexneri 2a**. *BMC Genomics* 2006, **7**:173.

33. Onodera NT, Ryu J, Durbic T, Nislow C, Archibald JM, Rohde JR: **Genome sequence of Shigella flexneri serotype 5a strain M90T Sm**. *Journal of bacteriology* 2012, **194**(11):3022.
